# Growth Stages Classification of Potato Crop Based on Analysis of Spectral Response and Variables Optimization

**DOI:** 10.3390/s20143995

**Published:** 2020-07-17

**Authors:** Ning Liu, Ruomei Zhao, Lang Qiao, Yao Zhang, Minzan Li, Hong Sun, Zizheng Xing, Xinbing Wang

**Affiliations:** 1 Key Laboratory of Modern Precision Agriculture System Integration Research, Ministry of Education, China Agricultural University, Beijing 100083, China; ningliu@cau.edu.cn (N.L.); S20193081422@cau.edu.cn (R.Z.); 13240948776@163.com (L.Q.); zhangyao@cau.edu.cn (Y.Z.); limz@cau.edu.cn (M.L.); S20183081362@cau.edu.cn (Z.X.); xbwang2020@cau.edu.cn (X.W.); 2 Key Laboratory of Agricultural information acquisition technology, Ministry of Agriculture and Rural Affairs, China Agricultural University, Beijing 100083, China

**Keywords:** precision agriculture, continuous wavelet transform (CWT), successive projection algorithm (SPA), random frog (RF), support vector machine (SVM)

## Abstract

Potato is the world’s fourth-largest food crop, following rice, wheat, and maize. Unlike other crops, it is a typical root crop with a special growth cycle pattern and underground tubers, which makes it harder to track the progress of potatoes and to provide automated crop management. The classification of growth stages has great significance for right time management in the potato field. This paper aims to study how to classify the growth stage of potato crops accurately on the basis of spectroscopy technology. To develop a classification model that monitors the growth stage of potato crops, the field experiments were conducted at the tillering stage (S1), tuber formation stage (S2), tuber bulking stage (S3), and tuber maturation stage (S4), respectively. After spectral data pre-processing, the dynamic changes in chlorophyll content and spectral response during growth were analyzed. A classification model was then established using the support vector machine (SVM) algorithm based on spectral bands and the wavelet coefficients obtained from the continuous wavelet transform (CWT) of reflectance spectra. The spectral variables, which include sensitive spectral bands and feature wavelet coefficients, were optimized using three selection algorithms to improve the classification performance of the model. The selection algorithms include correlation analysis (CA), the successive projection algorithm (SPA), and the random frog (RF) algorithm. The model results were used to compare the performance of various methods. The CWT-SPA-SVM model exhibited excellent performance. The classification accuracies on the training set (*A_train_*) and the test set (*A_test_*) were respectively 100% and 97.37%, demonstrating the good classification capability of the model. The difference between the *A_train_* and accuracy of cross-validation (*A_cv_*) was 1%, which showed that the model has good stability. Therefore, the CWT-SPA-SVM model can be used to classify the growth stages of potato crops accurately. This study provides an important support method for the classification of growth stages in the potato field.

## 1. Introduction

Precision agriculture is a central issue and management strategy for improved resource use efficiency, productivity, quality, and sustainability of agricultural production. [1] It is based on the variability estimation and decision-making with “right time, the right amount, and right place.” [2] Potato (Solanum tuberosum) is the world’s fourth-largest food crop, following rice, wheat, and maize. Unlike other crops, it is a typical root crop with a special growth cycle pattern and underground tubers [3,4], which makes it harder to track the progress of potatoes and to provide automated crop management. Therefore, the classification and variability estimation of growth stages is a critical step for making the right decision at the right time and guiding the automatic management of fertilizer or irrigation in the field. 

In agronomy, after sprouts, the potato growing cycle is in four main stages, which include the vegetative stage or tillering stage (S1), tuber formation stage (S2), tuber bulking stage (S3), and tuber maturation stage (S4) [5,6]. Observers have drawn attention to the closed relations between the canopy features and the development of underground tubers in the life cycle. Generally, the above-ground leaves, stems, and root system of the plant start to grow, and photosynthesis, which provides nourishment for the growing plant, begins at S2. Nutrient transfers to the underground tuber occur at S3. Accordingly, the demand for fertilizers and irrigation sharply increases from S2 to S3 to improve the quality and quantity of tubers [7,8]. This means the canopy information of the above-ground stems and leaves could help recognize the growing stages of potato plants. 

In recent reports, there is a primary concern with using spectroscopy in crop growth monitoring because of the principle of light absorption by molecular or chemical bonding [9,10]. Considerable literature focused on the reflected intensity and absorption location of sensitive wavelengths to detect the chlorophyll or water content during the growth stages [11,12]. Sun [13] analyzed the spectral migration characteristic of winter wheat at the jointing, booting, flowering, and milk-ripening stages. The wavelengths at red edge positions were extracted because of the capability to establish the chlorophyll detection models. Considering the difference in the canopy structure of barley crop at different growth stages, Yu [14] analyzed the spectral parameters to evaluate the chlorophyll content including the reflectance difference (RD), reflectance ratio (RR), normalized RD, the difference in RR, and the ratio of RD. The result showed that the RR could effectively eliminate the influence of structural differences on the canopy spectra. The relationship between water content and reflectance spectra of wheat was discussed at different growing stages in Graeff [15]. It was found that the bands of 490, 510, 540, 780, and 1300 nm could inhibit the influence of growth stages on the detection accuracy. Further, other reflectance vegetation indices were developed to monitor the crop growth states, for instance, the normalized difference vegetation index [16] was used to estimate the nutrition state; the chlorophyll index [17] and the structural independent pigment index [18] were used to predict the leaf pigment content; the photochemical reflectance index [19] was used to reflect the photosynthesis capability of crops; and the water index [20] was used to detect leaf moisture. However, these reflectance indices could not comprehensively reflect the growth stage information. 

The studies mentioned above outline the capability of the parameters estimating by spectroscopy, especially in the visible and near infrared band 320–1100 nm, which has sensitive responses to the physiology and biochemistry of vegetation [21] as well as the changes led by growth stages. Lots of methods have been used in data processing, especially in the exploration of variables to establish the inversion model, in which the independent variable is usually the wavelength of the light or unit directly proportional to the photon energy [22]. The sensitive wavelengths or specific vegetation indices are analyzed by multiple algorithms to classify the growth stages or evaluate the nutrient status [23]. In contrast to correlation analysis, several algorithms were proposed to reduce the problems of multicollinearity between adjacent wavelengths, such as principle component analysis, Monte Carlo uninformative variable elimination [24], competitive adaptive reweighted sampling [25], random frog (RF) [26], and successive projection algorithm (SPA) [27]. The RF and SPA were usually used to select sensitive wavelengths of crop growth parameters [28,29]. 

In contrast to the method based on location and optical intensity at a specific wavelength, continuous wavelet transformation (CWT) has an outstanding quality of a time and frequency domain, which can decompose a spectrum into several wavelet features (WFs) to characterize signals effectively [30]. Previous studies [31,32,33] reported that continuous wavelet analysis achieved good performance on crop estimation. Dong [32] indicated that WFs under 2^3^, 2^4^, and 2^5^ scales (middle- and low-frequency scales) could accurately detect the leaf nitrogen content of wheat and rice crops. The detection accuracy was higher than the normalized difference vegetation index. Lu et al. [34] indicated that the sensitive WFs of stripe rust and powdery mildew of wheat were distributed in 2^2^, 2^3^, and 2^4^ scales, and the WFs could capture the pigment and water content in the leaf of wheat. The above studies pointed out that the WFs under middle- and low-frequency scale factors can capture the peak and valley of an absorption feature of physical and chemical materials. However, the high-frequency WFs would remove the absorption features and could not detect the content of physiological and biochemical substances efficiently [33]. 

Previous studies combined the spectroscopy and algorithms to detect the nutrient content of potato canopy, for instance, Clevers [35] inverted the dynamic variation in the leaf area index (LAI) and chlorophyll content of potato canopy, Cohen [36] established the potato leaf chlorophyll content PLSR estimation model using canopy spectral data, and Ning [22] detected the water content of potato leaves. However, much uncertainty still exists about the relationship between reflected spectra from the canopy and the dynamic stages of the growing potatoes. Regarding the dynamic monitoring of the potato-growing stages, [37] previously proposed an algorithm to find the sensitive response parameters based on the variance analysis combined with variance reduction (VACVR). The results showed that these variables selected by VACVR based on reflectance spectra could classify the potato growth stages to a certain extent. However, their findings are inadequate for dynamic monitoring and classification of the potato growth cycle. 

Therefore, this paper aims to study how to classify the growth stage of potato crops accurately based on spectroscopy technology. This paper is organized into three parts. The first part discusses the dynamic response of chlorophyll content and canopy spectra in the growing stages. The second part shows how to find and extract the sensitive variables of growth stages by comparing different algorithms. Finally, the third part is how to establish the growth stage classification model. It will provide a spectroscopy mechanism explanation of valuable features extracted from potato canopy for the right time management in the field. 

## 2. Materials and Methods

**Figure 1:**
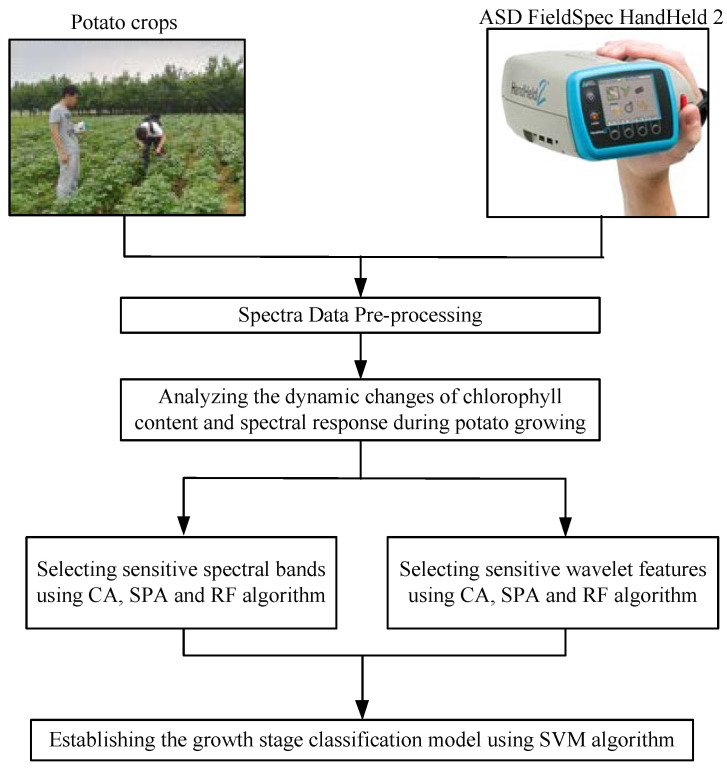
Flow chart of data analysis.

We conducted experiments and processed the data following the process flow shown in Figure 1. The main steps consist of spectra pre-processing, analysis of spectral response as the potato is growing, spectral variable optimization, and establishment of the classification model. There were four growth stages. In order to analyze data and establish the classification model, growth stages were represented by different labels. In this paper, the labels of the S1, S2, S3, and S4 stages are the numbers of 1, 2, 3, and 4. In the data analysis, the relationships between growth stage labels and spectral data were analyzed and discussed. 

### 2.1. Spectral Data Collection

The experiments were conducted at the national precision agriculture experiment station in Xiaotangshan town, Beijing, China, from May to June 2018. The growth stages of potato include the tillering (S1), tuber formation (S2), tuber expansion (S3), and tuber maturation (S4) stages. There was a total of 80 plots with a size of 1 × 1 m. There were about four potato plants per sampling plot. The experimental field had medium fertility. The cultivar of the potato crop was Atlantic. Details about the potato crop growth stages and sampling dates are shown in Table 1. 

Three leaves that were at the plant canopy were randomly collected per plot in the sampling field. Their reflectance spectra were measured by using an ASD FieldSpec-HandHeld-2 (Analytical Spectral Devices, Boulder, CO, USA), whose light source is sunlight. The spectrometer collects data at a 1 nm sampling interval in the range of 325–1075 nm. The experiment was conducted on cloudless and sunny days from 9 a.m. to 12 a.m. on May 15, May 24, June 7, and June 19. During data collection, the ASD device was located directly above the sample leaf, and the vertical distance from sensor to leaf was about 30 cm. The spectral reflectance was corrected by a standard calibration whiteboard every 10 min to eliminate the interference of variation in the solar illumination intensity spectral data. Reflectance data were taken from the three leaves, and the average value was calculated to represent the sampling spectrum in each plot. In data analysis, the band at 400–1000 nm was used because of the “fingerprint spectrum” phenomenon, which causes serious vibration noises in the 325–399 nm and 1001–1075 nm. 

### 2.2. Chlorophyll Content Measurement

The sampling leaves were packed and brought back to determine the chlorophyll content in the laboratory. Each potato leaf was cut into pieces. The 0.04 g pieces of each leaf were put into 25 mL mixture of acetone and anhydrous ethanol to extract chlorophyll. The volume ratio of acetone and anhydrous ethanol was 2:1. The extraction solution was placed in the dark for 24 h. Then the absorbance of the extraction solution was measured using a UV-752 spectrophotometer (Shimadzu, Japan) at 645 and 663 nm. According to previous spectrophotometric analysis of chlorophyll from an extraction solution [38], the chlorophyll content was measured following the equations

(1) $$C_{a}=12.72A_{663}-2.59A_{645}$$

(2) $$C_{b}=22.88A_{645}-4.67A_{663}$$

(3) $$C_{t}=C_{a}+C_{b}$$

where $A_{645}$ and $A_{663}$ are respectively the absorbance at 645 and 663 nm, $C_{a}$ and $C_{b}$ are respectively the content of chlorophyll-a and chlorophyll-b, and $C_{t}$ is the total chlorophyll content. 

The average chlorophyll value of three leaves per plot was calculated to represent the chlorophyll content of this plot sample. There were 80 groups of data collected in each growth stage, in which six groups were invalid because of the influence of low vegetation coverage, so 74 groups data were retained in S1. Reflectance spectra for a total of 314 groups were obtained. 

### 2.3. Pre-Processing of Spectral Data

After correcting reflectance with a standard calibration whiteboard, the spectral reflectance curves still have polynomial baseline shifts and noises combined by several additives and multiplicative factors caused by the different light reflection paths under the field measurement condition [39]. Moreover, interference is often difficult to separate from reflection spectra. The original reflectance spectra were pre-processed by the standard normal variate (SNV) to eliminate the noise. SNV is a certified method that can remove both additive and multiplicative effects in spectral data [40]. In SNV, each spectrum is centered and then scaled by the corresponding standard deviation. It could be calculated with Equation (4):

(4) $$z_{i}=\frac{x_{i}-\mu }{\sigma }$$

where $x_{i}$ is the reflectance of i nm, μ is the average reflectance of a spectrum, σ is the standard deviation of a spectrum, and $z_{i}$ is the reflectance after SNV of i nm. 

### 2.4. Sample Subset Partitioning

The sample set partitioning based on the joint X-Y distance (SPXY) algorithm was applied to divide samples into the calibration and validation set. It consists of the Euclidean distance of the independent variable and a distance in the dependent variable space for the parameter under consideration [41]. Several published studies indicated that the performance of SPXY was better than the Kennard–Stone and the random selection methods, which was due to the fact that SPXY can comprehensively obtain the difference between the independent and dependent variables among samples. The training set partitioned by the SPXY algorithm can strongly represent the whole data set, which makes the performance of the trained model more stable [42]. Li [43] reported that it was suitable for training models to have the sample set divided according to the ratio of 2:1. In this research, the sample set (80 samples) was divided into a training set and a test set at each growth stage, so the division ratio of 5:3 was chosen. 

### 2.5. Continuous Wavelet Transformation

Mathematically, CWT is a linear operation that performs the convolution of the reflectance spectrum with a scaled and shifted parent wavelet. The transform process is shown in Equation (5):

(5) $$W_{f}(a,b)=\frac{1}{\sqrt{a}}\int_{-\infty }^{+\infty }f(\lambda )\psi (\frac{\lambda -b}{a})d\lambda$$

where, ψ(λ) is the parent wavelet function, f(λ) is the reflectance spectrum, and $W_{f}(a,b)$ is the wavelet coefficient (or WF, denoted as $WF_{a,b}$) for the scaling factor a and the shifting factor b. The scaling factor indicates the width of the scaled parent wavelet. The scaling factor used in this study is at dyadic scales $2^{n}(n=1,2,\ldots ,10)$, denoted as scale 1, scale 2, …, scale 10, etc. The shifting factor is the central wavelength of the shifted parent wavelet. The physical and chemical components of crops have characteristic spectral absorption. The b is used to capture the peak and valley of an absorption feature, and the scaling factor a is comparable with the absorption feature’s width. The second derivative of the Gaussian function served as the parent wavelet function in this study. 

The one-dimensional reflectance spectra are transformed into two-dimensional wavelet power map data composed of scaling factor (frequency scale) and shifting factor (spectral wavelength) by using the CWT. According to previous literature, the scaling factor from 1 to 3 belongs to low frequency. The scaling factor from 4 to 7 belongs to middle frequency, and the scaling factor from 8 to 10 is high frequency [31,32,33,34]. The sensitive spectral variables of potato growth stages can be extracted from these wavelet power coefficients. 

### 2.6. Variables Selection Algorithm

Since the spectrometer collects reflectance data based on near contiguous spectral bands, the selection of sensitive wavelengths or variables is one of the key steps in the chlorophyll detection to solve multiple mutual linear problems of overfitting and redundancy. Thus, the algorithms were used and compared between SPA and RF to select more representative and robust sensitive wavelengths. 

#### 2.6.1. Successive Projection Algorithm

The SPA proposed by Araújo [44] is a forward selection method to solve collinearity problems. It starts with one wavelength and then incorporates another one at each iteration until a specified number of wavelengths is reached. 

If the calibration set consists of M samples and J variable, the column vector is subjected to a sequence of projection operations that create J chains of K variables, where K is the minimum value between M-1 and J [45]. The SPA works in three steps: 

Step1: For each chain, select a variable randomly as the initial variable. Then, select a new variable from among all remaining variables, the selected variable has the maximum projection value of the previously selected variable; 

Step2: For each variable subset extracted based on Step1, a multiple linear regression (MLR) model is established, and the root mean square error of the validation set (RMSEV) is calculated. The subset whose RMSEV is the smallest is selected; 

Step3: Using a backward elimination procedure, discard redundant variables from the subset selected by Step2. 

Thus, the selected wavelengths by SPA contained minimally redundant information content. 

#### 2.6.2. Random Frog Algorithm

RF is a mathematically simple and computationally efficient method, which borrows the framework of reversible jump Markov chain Monte Carlo (RJMCMC) [46]. It searches the model space through both fixed-dimensional and trans-dimensional moves between different models. Then, a pseudo-RJMCMC chain is computed and used to calculate the selection probability of each variable. Afterwards, variables are selected based on the ranking of all variables. 

The algorithm of RF was fully detailed in [47]. Briefly, RF works in three steps: 

Firstly, a variable subset V_0_ containing M variables is initialized randomly; 

Secondly, a candidate variable subset V* including N* variables is proposed based on V_0_; accept V* with a certain probability as V_1_, and replace V_0_ using V_1_; 

Thirdly, repeat the second step until N iterations are finished. 

Then, compute a selection probability of each variable, which can be used as a measure of variable importance. 

In this paper, the number of iterations of RF was set as 1000. The average selection probability was obtained after 10 repetitions. The wavelengths with the selection probability greater than 0.25 were selected as chlorophyll sensitive wavelengths. 

### 2.7. Support Vector Machine Modeling Method

Support vector machine (SVM) is a smaller sample size learning algorithm, which shows great advantages in high-dimensional pattern recognition. It is valued and has been used by scholars in classification. SVM is based on the VC theory of statistical theory and the principle of structural risk. The basic idea is that SVM transforms the sample data into a high-dimensional feature space, and then constructs the optimal kernel function in the space to create a hyperplane. The greater the interval between hyperplanes, the better the classification effect [48]. 

In this paper, the radial basis function (RBF) serves as the SVM kernel function. In the RBF, two main parameters are affecting the SVM classifier, which are the setting parameter *g* and the penalty parameter *c* of the gamma function in the RBF. The grid search algorithm is used to optimize the parameters *g* and *c*. The search space of both parameters is 2^−10^ to 2^10^. Internal cross-validation was performed using 10-fold cross-validation, and parameters *g* and *c* were determined using the accuracy of cross-validation (*A_cv_*). 

The accuracy of the train set (*A_train_*), the difference between *A_train_* and *A_cv_* (*A_train_* − *A_cv_*), and the accuracy of the test set (*A_tes_*_t_) are calculated to evaluate the model performance. The value of *A_train_* − *A_cv_* is used to evaluate the model robustness, where a smaller *A_train_* − *A_cv_* value implies a more robust model. Furthermore, the *A_tes_*_t_ is applied to evaluate the model accuracy, and a high *A_tes_*_t_ value indicates a more accurate model with better classification capability. 

## 3. Results

### 3.1. Statistics on Chlorophyll Content and Sample Set Partition

**Figure 2:**
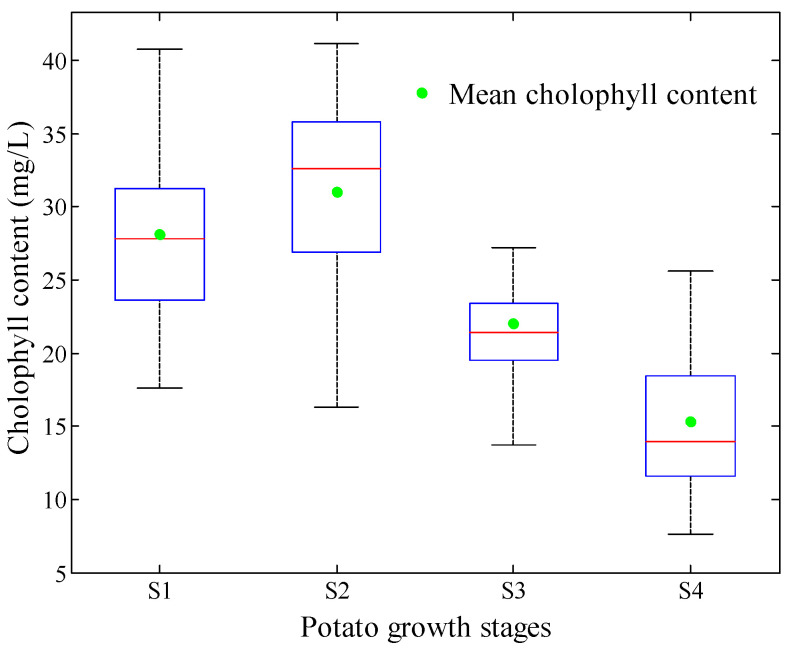
Statistical box line graph of chlorophyll content during potato growth stage.

The chlorophyll contents were measured from S1 to S4. The average value at each stage was calculated, which represents the dynamic changes at each growth stage. The results are shown in Figure 2. The chlorophyll contents increased from 28.12 at S1 to 31.04 mg/L at S2 and gradually decreased to 15.36 mg/L at S4. 

Table 2 shows the statistical description of the sample set for each growth stage and a combination of the data from all four stages. The samples from all growth stages were combined to represent the changes in chlorophyll content. The data set for the growth stage classification model consists of the training and test sets with 200 and 114 samples, respectively. It also can be seen that the maximum value of the training set (41.20) is bigger than that of test set (37.46), and the minimum value of training set (7.66) is smaller than that of test set (8.20). The result further reveals that the division result by SPXY was reasonable, and the training set could strongly represent the whole data set. 

### 3.2. Analysis of Spectral Response During Growth

**Figure 3:**
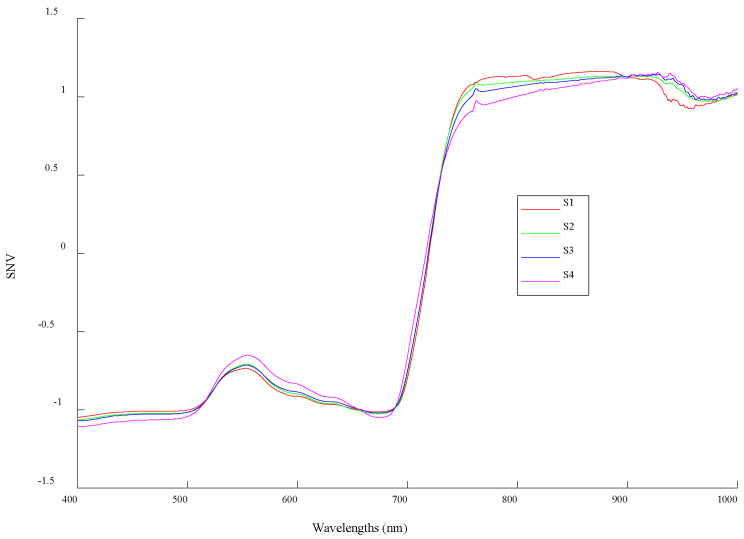
Average canopy spectral curve per potato growth stage after standard normal variate (SNV).

The spectral reflectance curves were analyzed after denoising using SNV correction, and the dynamic changes between different stages were analyzed based on the average spectrum of each stage. Figure 3 shows the reflectance of each stage. Their trends were similar in the visible region (400–760 nm) and the near-infrared region (761–1000 nm). In the visible region, the minimum reflectance appeared near 400 and 680 nm because of strong absorption by the pigment. In the near- infrared region, the reflectance sharply increased from 711 to 760 nm because the reflective surface cavity is in the spongy structure of the mesophyll. Although there was a strong reflection in 761–1000 nm as a horizontal platform, a weak reflectance valley appeared near 970 nm because of the weak absorption of leaf water content. 

However, there were significant changes in some specific bands during growth. In the bands of 530–640 nm, the spectral reflectance increased with growth. The average reflectance in S4 was significantly lower than in other stages of growth, whereas the average reflectance of S2 and S3 was very close. In the bands of 740–880 nm, the spectral reflectance decreased gradually, and there were small reflectance peaks near 763 nm in the S2, S3, and S4 stages. In the bands of 910–960 nm, the average value in S1 was significantly lower than in other stages of growth. 

### 3.3. Correlation between Chlorophyll Content and Reflectance Spectra

**Figure 4:**
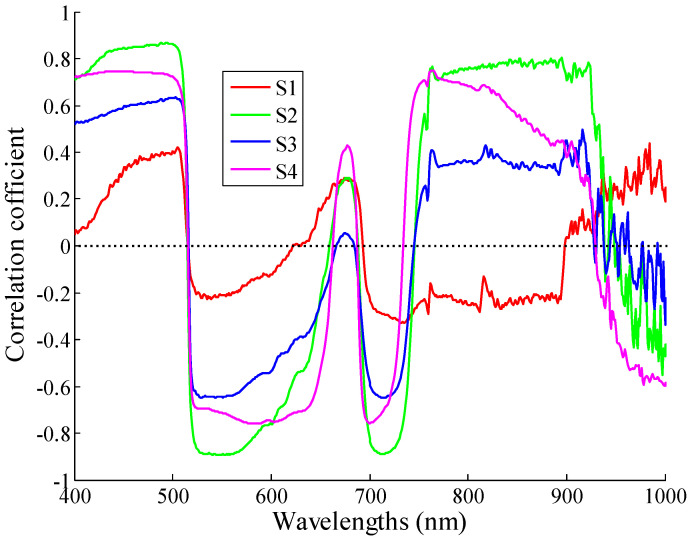
Correlation coefficient curve between chlorophyll content and reflectance spectra.

To further understand how the spectral reflectance changes with the potato growth, correlation analysis was conducted between spectral reflectance and chlorophyll content from S1 to S4. Figure 4 shows the correlation coefficient curves. The chlorophyll content correlated positively with the reflectance spectra in the ranges of 400–500 and 650–700 nm. However, there was a negative correlation between them in the ranges of 510–630 and 701–750 nm. Furthermore, four band regions were highly correlated, which included 400–510, 521–610, 701–740, and 761–920 nm. 

Overall, the correlation coefficients of S1–S4 had significant differences in the ranges of 400–600, 601–620, and 700–902 nm. Conversely, the curve trend of the correlation coefficients of S2 and S3 were very similar. The results of the analysis are shown in Table 3. The correlation values were listed in descending order in the ranges of 400–600 and 701–900 nm as |S2|, |S4|, |S3|, and |S1|, while these changed to |S4|, |S2|, |S3|, and |S1| in the range of 601–620 nm. 

### 3.4. Sensitive Wavelengths Selection for Dynamic Growth

#### 3.4.1. Correlation Analysis between Growth Stages and Reflectance Spectra

**Figure 5:**
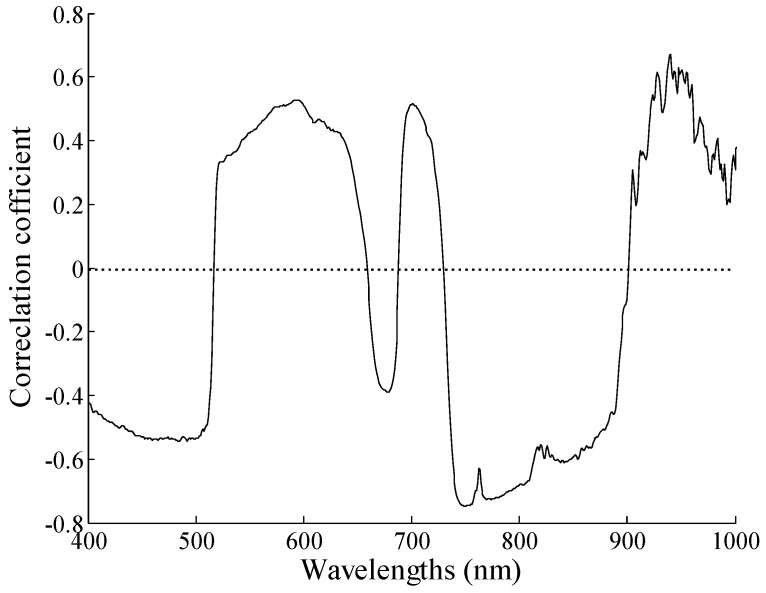
Correlation curve between growths stages and reflectance spectra.

Figure 5 shows the correlation between growth stages and reflectance. In the ranges of 430–510 and 735–880 nm, the reflectance spectra correlated negatively with the growth stages, and the absolute value of the correlation coefficients were higher than 0.5. The absolute values of the correlation coefficients were higher than 0.7 in the ranges of 742–758 and 765–790 nm. In the ranges of 570–600 and 920–960 nm, the reflectance spectra correlated positively with the growth stages, and the correlation coefficients were higher than 0.5. Furthermore, there were extreme correlation values near 492, 594, 678, 700, 750, and 940 nm, and the correlation coefficients were −0.544, 0.526, −0.391, 0.514, −0.748, and 0.671, respectively. The wavelength with the highest correlation was 750 nm, and the correlation was −0.748. 

The spectral bands with an absolute correlation coefficient value greater than 0.7 were selected as the growth stage-sensitive variables. Based on the correlation analysis (CA), a total of 40 wavelengths were selected and identified as the CA bands. In the selected CA bands, 17 bands were distributed in the range of 742–758 nm (red light region), and 23 bands were distributed in the range of 766–788 nm (near infrared region). Moreover, there is severe multi-collinearity in the CA bands. 

#### 3.4.2. Sensitive Wavelengths Selection Using SPA

**Figure 6:**
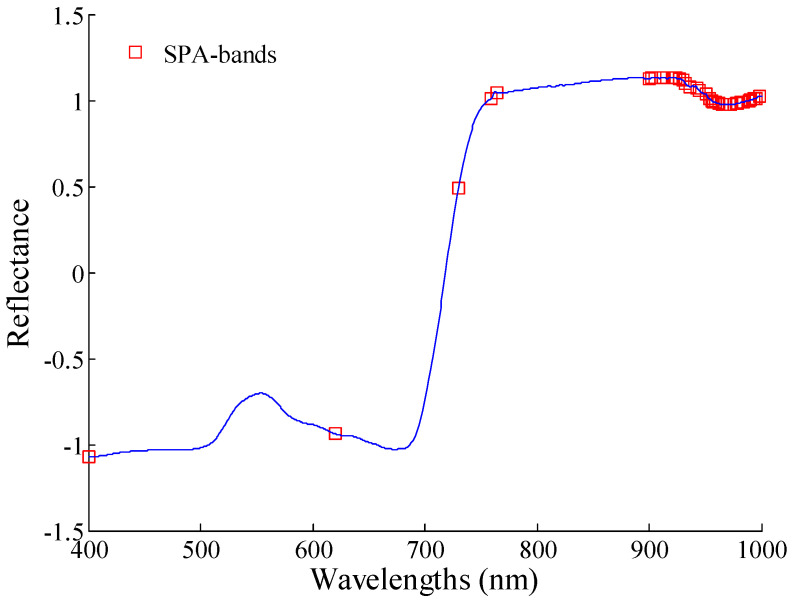
Growth stage-sensitive wavelengths selection result of potato using the successive projection algorithm (SPA).

In the range of 400–1000 nm, 36 growth stage-sensitive wavelengths were selected using SPA, denoted as SPA bands. The location of the SPA bands is shown in Figure 6. Four sensitive wavelengths were in the visible region (400–760 nm), which included 400, 620, 730, and 759 nm. There were 32 sensitive wavelengths in the near-infrared region (761–1000 nm). Besides 764 nm, there were 31 sensitive wavelengths in 900–1000 nm, with a “wavelength aggregation” phenomenon. Moreover, there was severe multi-collinearity and a large amount of redundant information among the 31 sensitive wavelengths. 

#### 3.4.3. Sensitive Wavelengths Selection Using RF

**Figure 7:**
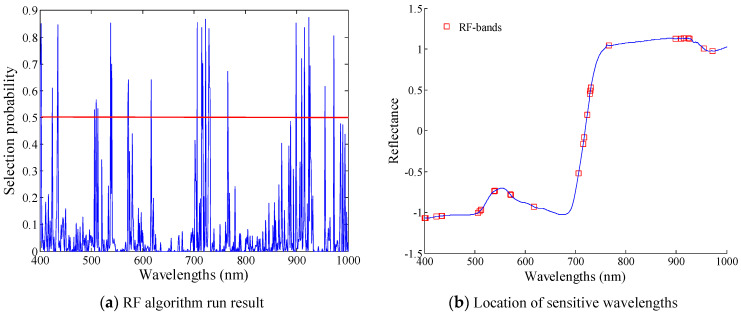
Growth stage-sensitive wavelengths selection result of potato using SPA.

The number of iterations the RF algorithm was set to was 1000. The average result was computed after 10 repetitions. Figure 7a shows the result within 400–1000 nm, where the *y*-axis is the probability that each wavelength will be selected as the sensitive wavelength. The wavelengths with a probability greater than 0.5 were selected as the growth stage-sensitive wavelengths to establish the growth stage classification model. 

Using the RF algorithm, 29 sensitive wavelengths were selected, which were denoted as the RF bands. The RF bands included 401, 402, 403, 424, 434, 435, 506, 510, 513, 538, 540, 571, 572, 617, 706, 715, 717, 723, 729, 730, 731, 766, 899, 910, 915, 924, 926, 955, and 972 nm. Figure 7b shows the location of RF bands. 

In the visible region (400–760 nm), there were 21 sensitive wavelengths, which reflected the information of complex pigment macromolecules in potato leaves such as chlorophyll and carotenoid. Alternatively, in the near-infrared region (711–1000 nm), there were eight sensitive wavelengths, which could reflect the structural information of potato leaves. 

Comparing the RF bands with the SPA bands, the RF bands contained more sensitive wavelengths in the visible light region. Moreover, the wavelengths distribution was uniform in the near infrared region. 

### 3.5. Sensitive Spectral Variables Selection Based Continuous Wavelet Analysis

#### 3.5.1. Correlation Analysis between Growth Stages and Wavelet Features

**Figure 8:**
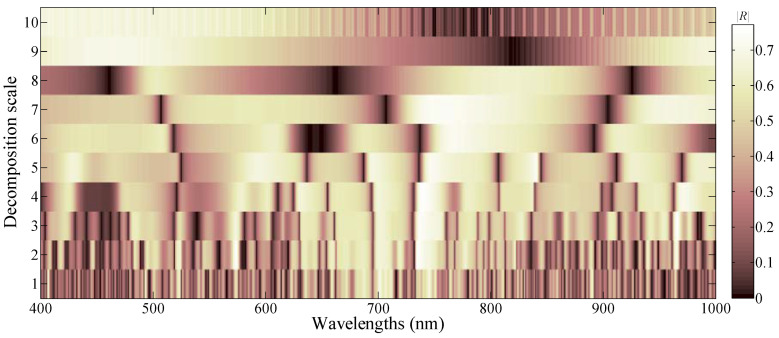
Correlation coefficients map between wavelet features and growth stages.

The correlation coefficients between the growth stages and WFs were calculated under 10 scaling factors. The correlation relation distribution map was drawn based on the absolute value of correlation coefficients. Figure 8 reveals that the correlation coefficients varied under different scales (scaling factors) and wavelength locations (shifting factors). Further, the WFs with high correlation (|R|>0.6) distributed in the range of 400–500 nm under scale 9 and scale 10, around 570 nm under scale 2, scale 3, and scale 4, around 600 nm under scale 5 and scale 6, around 700 nm under scales 1–6, around 740 nm under scales 2–6, in the range of 750–780 nm under scales 6 and 7, in the range of 930–1000 nm under scale 7, in the range of 960–970 nm under scales 2–4, and in 980–1000 nm under scale 5. 

Using CA, 1% of total 6010 WFs were selected as sensitive variables, which were denoted as CA-WFs. The CA-WFs consist of WFs with absolute correlation values greater than 0.75. The results are shown in Table 4. 

Based on the scaling factor, the CA-WFs were all distributed all in the middle- (scales 4–6) and low-frequency (scales 1–3) scales. Conversely, based on the shifting factor, CA-WF1 was distributed in the green light region. At the same time, CA-WF2, CA-WF3, CA-WF5, CA-WF6, CA-WF8, and CA-WF9 are located in the red-light region. The WFs in the visible region reflected the leaf pigment, such as chlorophyll. Furthermore, the CA-WF4 and CA-WF7 located in the near-infrared region reflected the leaf structure and other substances, for instance, the CA-WF4 near 970 nm reflected the leaf water content. 

Similar to the CA bands, the CA-WFs had severe multi-collinearity. For example, the CA-WF9 contained 21 WFs, which were distributed continuously in the range of 741–761 nm. The SPA and RF algorithm were used to select growth stage-sensitive WFs to reduce the redundant information and to improve the performance of sensitive variables. 

#### 3.5.2. Selection of Sensitive Wavelet Features Using SPA

**Figure 9:**
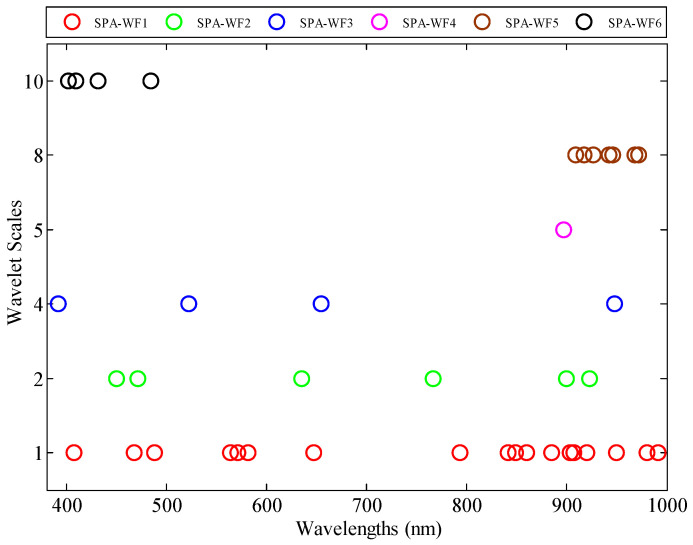
The location of growth stage-sensitive wavelet features selected by SPA.

SPA was used to select 40 WFs, which are categorized into six frequency scales, denoted as SPA-WFs. Figure 9 shows the distribution of SPA-WFs. 

Based on the scaling factor, SPA-WF1 (scale 1) and SPA-WF2 (scale 2) were distributed in the low-frequency scales. The SPA-WF3 (scale 4) and SPA-WF4 (scale 5) were distributed in middle-frequency scales. The SPA-WF5 (scale 8) and SPA-WF6 (scale 10) were distributed in high-frequency scales. Unlike the CA-WFs, which were distributed in middle- and low-frequency scales, SPA-WFs were distributed in low-, middle-, and high-frequency scales. The high-frequency WFs possessing a wide-scale window could smooth the reflectance spectra, but which would remove some absorption features. 

Based on the shifting factor, the seven WFs of SPA-WF1 and three WFs of SPA-WF2 and SPA-WF3 were located in the blue-, green-, and red-light regions, which reflected the leaf pigment information when distributed in low-frequency scales. The 11 WFs of SPA-WF1, the three WFs of SPA-WF2, one WF of SPA-WF3, and SPA-WF4 located in the near-infrared region reflected other leaf substances; for instance, the SPA-WF_1,907_ and SPA-WF_1,909_ near 909 nm reflected the leaf protein, and the SPA-WF_4,964_ near 970 nm reflected the leaf water. The seven WFs of SPA-WF5 (scale 8) and four WFs of SPA-WF6 (scale 10) were located in the ranges of 900–1000 and 400–440 nm, respectively, which could not reflect any leaf information when distributed in high-frequency scales. 

#### 3.5.3. Selection of Sensitive Wavelet Features Using RF

**Figure 10:**
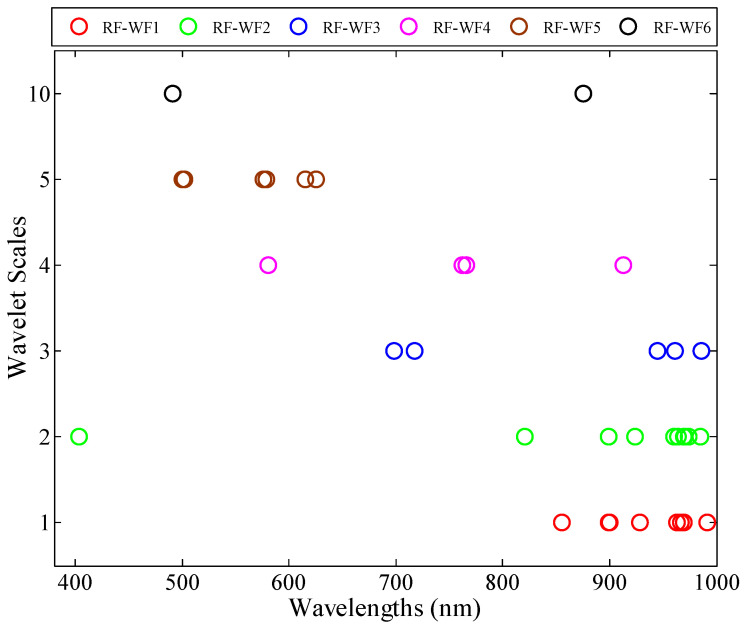
Location of growth stage sensitive wavelet features selected by random frog (RF).

The 36 WFs were selected using RF, which included also six frequency scales, denoted as RF-WFs. The distribution of RF-WFs is shown in Figure 10. 

Based on the scaling factor, RF-WF1 (scale 1), RF-WF2 (scale 2), and RF-WF3 (scale 3) were distributed in the middle- and low-frequency scales. The RF-WF4 (scale 4) and RF-WF5 (scale 5) were distributed in middle-frequency scales. The RF-WF6s (scale 10) were distributed in high-frequency scales. The number of high-frequency WFs was less than SPA-WFs, but more than CA-WFs. 

Based on the shifting factor, a total of four WFs in RF-WF1, RF-WF2, RF-WF3, and RF-WF4 were located in the visible light region, which included RF-WF_2,400_, RF-WF_3,690_, RF-WF_3,710_, and RF-WF_4,587_. Moreover, six WFs of RF-WF5 were located in the visible region. Therefore, the RF-WFs compared with SPA-WFs had a weaker ability to reflect the leaf pigment. Furthermore, nine WFs of RF-WF1 and RF-WF2, the three WFs of RF-WF3, and RF-WF4 were located in the near-infrared region. RF-WF_1,968_ and RF-WF_1,970_ could reflect the leaf water, whereas RF-WF_1,992_, RF-WF_2,990_, and RF-WF_3,989_ near 992 nm reflected the leaf starch. The two WFs of RF-WF6 (scale 10) were RF-WF_10,493_ and RF-WF_10,876_. 

### 3.6. Establishing Growth Stage Identification Model Using SVM

The growth stage classification models were established respectively based on sensitive spectral bands and sensitive WFs by using the SVM algorithm. Moreover, the *g* and *c* parameters of the SVM kernel function were optimized using the grid search algorithm. Table 5 shows the results of the kernel function parameters optimization and model establishment. 

**Figure 11:**
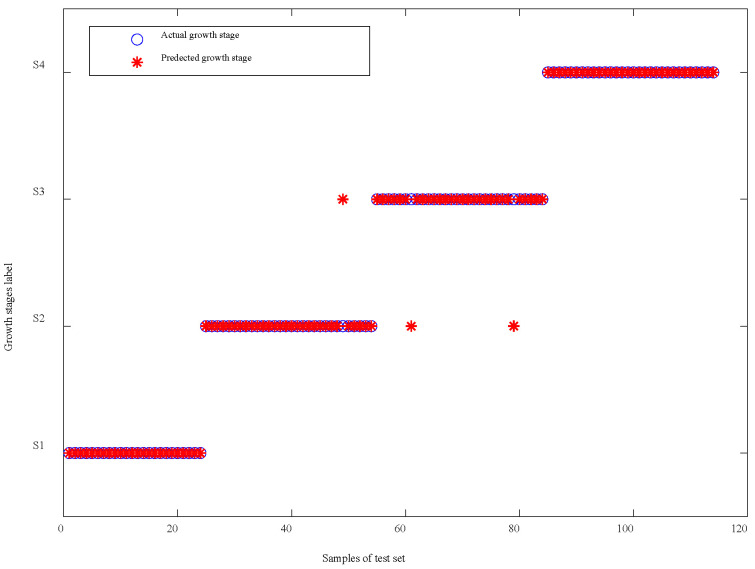
Comparison chart between the actual growth stage and identification results of the test set.

The value of (*A_train_* − *A_cv_*) represents the robustness of each model. Therefore, sorting the model in ascending order based on the value of (*A_train_* − *A_cv_*) showed that the SPA-CWT-SVM model is the most robust, whereas the CA-SVM model is the least robust. Furthermore, model accuracy is represented by the value of *A_test_.* Arranging the model in descending order based on the value of *A_test_* revealed that the SPA-CWT-SVM model had the highest classification capability. Conversely, the CA-SVM model had the lowest classification capability. We compared the accuracy of models on the basis of sensitive wavelengths and sensitive WFs selected by the same algorithm. The accuracies of the model based on WFs CA-CWT-SVM (92.11%), SPA-CWT-SVM (97.37%), and RF-CWT-SVM (94.74%) were higher than the model based on bands CA-SVM (86.84%), SPA-SVM (92.11%), and RF-SVM (94.59%). Therefore, the SPA-CWT-SVM model was selected to classify the growth stages of potato crops. The performance of the model on the test set is shown in Figure 11. 

Further analyzing the wrongly classified samples of the test set, there was a total of three wrongly classified samples. The three wrongly classified samples were all in S2 and S3, while there was none in S1 and S4. The reason was that both S2 and S3 were in the growth stages when the nutrient was being transported from stems and leaves (above ground part) to the tuber (underground part), so the canopy structure of S2 and S3 was relatively similar. Although the chlorophyll content reduced however, there was no significant difference in the canopy spectra. 

## 4. Discussion

Spectroscopy is a non-destructive method for the detection of crop phenotype information such as canopy structure, plant sharp, and stress status [49,50], and physiology status such as pigment, moisture, and disease [51,52]. The physical and chemical substances, leaf tissue, and canopy structure change as the potato grows. Consequently, the spectral characteristics reflect the changes in physiological and biochemical composition and canopy structure of crops [53]. Therefore, the reflectance spectra data can be applied to classify the potato crop growth stages. 

In this study, the spectral characteristics response and chlorophyll content change at different stages were analyzed and discussed. The results demonstrated that average reflectance was close in S2 and S3, and the correlation curves between the reflectance and chlorophyll content of S2 and S3 had similar change trends. According to the potato phenology, the new tuber forms by stolons after the plant flowers at S2 and the tuber expands at S3. Consequently, the nutrient availability and balance are transferred from above-ground stems and leaves to underground tubers during these periods. This phenomenon might explain why some of the plants have similar physiology statuses and spectral responses [5]. 

The correlation coefficients between spectral wavelengths, WFs, and growth stages were calculated, where the correlation coefficients of 40 spectral wavelengths are higher than 0.7. The correlation coefficients of at least 60 WFs are higher than 0.75, which illustrates that the CWT could enhance the correlation of growth stages by decomposing spectral data. Previous studies reported the same results, such as Wang [54], who indicated that the correlation between wavelet coefficients and pigments was significantly higher than that of the vegetation index and bands. However, both CA bands and CA-WFs had severe multi-collinearity, which resulted in the poor performance of the CA-SVM and the CA-CWT-SVM models. Therefore, the spectral bands and wavelet coefficients were further optimized by using the SPA and RF algorithm to establish a high-performance growth stage classification model. 

The SPA is an effective algorithm to solve collinearity problems [44]. Many researchers applied SPA to select sensitive spectral variables and to improve model performance. Moreover, RF, developed based on the model population analysis strategy [47], can be used to consider the contribution of each variable to the target property to select informative spectral variables. The SPA (a forward selection method) and RF (a backward selection method) have their advantages. In this paper, SPA and RF were utilized to select the sensitive growth stage spectral variables (including bands and WFs). First, the SPA and RF were used to select sensitive bands, which was applied to establish the classification model. The RF-SVM model achieved the best classification accuracy, followed by SPA-SVM, and CA-SVM was worst, which proved that the RF algorithm was effective for selecting sensitive wavelengths of the growth stages. Furthermore, for the selection of sensitive spectral variables, the SPA and RF was used again to select sensitive WFs after spectra CWT. The test set accuracies (*A_test_*) of classification models based on sensitive WFs CA-CWT-SVM (92.11%), SPA-CWT-SVM (97.37%), and RF-CWT-SVM (94.74%) were higher than the model based on bands CA-SVM (86.84%), SPA-SVM (92.11%), and RF-SVM (94.59%). The results illustrated that the CWT could dig deeper into the spectral information variables. Furthermore, the *A_test_* of SPA-CWT-SVM (97.37%) was higher than RF-CWT-SVM (94.74%), which demonstrated that the SPA was suitable for selecting sensitive WFs. 

Relative to the full spectral bands of 601 variables and full wavelet coefficients of 6010 variables, the numbers of sensitive variables (bands and WFs) selected by SPA and RF were reduced significantly. However, there were still more variables in the classification models. Over-fitting frequently occurred during the modeling process, caused by the increasing number of model variables, which affects the stability and accuracy of the classification model [55]. Therefore, internal cross-validation was performed in this study. The difference in the accuracy of the training set (*A_train_*) and accuracy of the cross-validation (*A_cv_*, *A_train_* − *A_cv_*) is used as an indicator to determine the stability of the model. Compared with other classification models, SPA-CWT-SVM (*A_train_* − *A_cv_* = 1.00%) was the most stable model. 

It is worth noting that both SPA and RF selected the high-frequency WFs, such as SPA-WF5 (scale 8), SPA-WF6 (scale 10), and RF-WF6 (scale 10). Moreover, SPA-WF5 and SPA-WF6 included 11 sensitive variables, and the RF-WF6 included only two sensitive variables. Previous literature reported that the WFs under middle- and low-frequency scales could capture the absorption characteristics of physical and chemical substances of crops [31,34]. The high-frequency WFs would remove the absorption features and, therefore, could not efficiently detect the physiological and biochemical compositions [56]. However, this study revealed that high-frequency WFs could remove the high-frequency noise of reflectance spectra and sharpen the difference in spectra. It illustrated that high-frequency WFs could play a unique role in qualitative analysis compared with quantitative inversion analysis. The result provides a reference for using CWT in classification research based on spectral data. 

A comprehensive analysis of the above results showed that the spectral data were processed using the CWT. Moreover, the sensitive variables were selected using the SPA, which was suitable for model variables optimization and classification accuracy improvement. The above data analysis method (SPA-CWT-SVM) has promising potential for classifying the growth stages of potato crops. However, the method in this study is based on specific spectral data for potato crops. The restrictions are based on the existence of other data sets or potato varieties [57]. Therefore, more data sets from a wide range of potato varieties, planting patterns, and experimental fields should be collected to develop a stable and accurate classification model using the SPA-CWT-SVM method. 

## 5. Conclusions

This paper has presented the potential and the methods for monitoring the growth stages of potato plants using canopy spectroscopy. The dynamic responses of canopy spectra at different growth stages were analyzed. The spectral characteristics were significantly different between S1, S2–S3, and S4. However, the spectral reflectance curves in S2 and S3 were similar. The CWT was used to decompose one-dimensional spectra under different frequency scales into two-dimensional WFs to establish the high-performance classification model of potato crop growth stages. The *A_test_* of classification models based on WFs CA-CWT-SVM (92.11%), SPA-CWT-SVM (97.37%), and RF-CWT-SVM (94.74%) were higher than the classification models based on bands CA-SVM (86.84%), SPA-SVM (92.11%), and RF-SVM (94.59%). The results proved that the CWT could dig deeper into the spectral information variables, which enhanced the classification accuracy of the growth stages. The SPA-CWT-SVM model performed better than other models. Based on spectral data, the SPA-CWT-SVM is a potentially accurate and efficient model to monitor the growth stage of potato crops. This research explored an automatic method to indicate the specific stage of growing potatoes. However, further experiments need to be conducted to prove the performance of the proposed model. 

## Figures and Tables

**Table 1:** The information about potato crop samples.

Growth Stage	Potato Crop Characteristics	Collection Date	Samples
S1	Appearing flower buds, having about 12 leaves	15 May	74
S2	Appearing flowers	24 May	80
S3	Flowers falling, stems and leaves aging, and the lower leaves being yellow	7 June	80
S4	Stems and leaves withering, and the upper leaves being yellow	19 June	80

**Table 2:** Chlorophyll content statistics of training and test sets (mg/L).

Samples	Data Set	Sample Number	Max	Min	Mean	SD
S1	all	74	40.77	17.64	28.12	5.05
	train	50	40.77	17.64	28.27	5.31
	test	24	33.12	19.64	27.48	3.86
S2	all	80	41.20	16.30	31.04	5.81
	train	50	41.20	16.30	30.23	6.29
	test	30	37.46	25.26	33.45	3.04
S3	all	80	35.63	13.70	22.00	4.18
	train	50	35.63	13.70	22.04	4.65
	test	30	26.47	16.39	21.86	2.36
S4	all	80	32.25	7.66	15.36	5.45
	train	50	32.25	7.66	15.73	5.93
	test	30	20.69	8.20	14.24	3.55
All stages	all	314	41.20	7.66	24.05	7.95
	train	200	41.20	7.66	24.07	7.95
	test	114	37.46	8.20	24.00	8.00

**Table 3:** The analysis results of correlation difference in some bands.

Bands Region	Correlation Order	Negative Correlation
400–510	S2 > S4 > S3 > S1	None
511–600	|S2| > |S4| > |S3| > |S1|	All
601–620	|S4| > |S2| > |S3| > |S1|	All
701–750	|S2| > |S4| > |S3| > |S1|	All
751–900	S2 > S4 > S3 > |S1|	S1

**Table 4:** The information of wavelet features selected using correlation analysis.

Wavelet Feature	Scale	Wavelengths	*|R|*
CA-WF1	2^1^	572–573(2)	>0.71
CA-WF2	2^1^	734–737(4)	
CA-WF3	2^2^	735–741(7)	
CA-WF4	2^2^	964–966(3)	
CA-WF5	2^3^	736–745(10)	
CA-WF6	2^4^	745–749(5)	
CA-WF7	2^4^	839	
CA-WF8	2^5^	763–769(7)	
CA-WF9	2^6^	741–761(21)	

**Table 5:** Statistics of potato growth stage classification model.

Model	Variables	g	c	*A_cv_* (%)	*A_train_* (%)	*A_train_* − *A_cv_* (%)	*A_test_* (%)
CA-SVM	40	0.25	1024.00	78.00	88.50	10.50	76.32
SPA-SVM	36	0.03	337.79	90.50	99.00	8.80	92.11
RF-SVM	29	0.045	1024.00	96.25	98.75	2.50	94.59
CA-CWT-SVM	75	0.14	194.01	90.00	97.50	7.50	92.11
SPA-CWT-SVM	40	0.63	71.46	99.00	100.00	1.00	97.37
RF-CWT-SVM	36	0.44	36.75	94.00	99.50	5.50	94.74
